# As air relative humidity increases, infectivity of SARS-CoV-2 decreases within water droplets

**DOI:** 10.1017/qrd.2024.7

**Published:** 2024-08-29

**Authors:** Yu Liu, Lei Cao, Yu Xia, Pan Pan, Lang Rao, Bolei Chen, Richard N. Zare

**Affiliations:** 1 Institute of Biomedical Health Technology and Engineering, Shenzhen Bay Laboratory, Shenzhen, P. R. China; 2 Hubei Key Laboratory of Environmental and Health Effects of Persistent Toxic Substances, School of Environment and Health, Jianghan University, Wuhan, P. R. China; 3 Department of Chemistry, Stanford University, Stanford, CA, USA; 4 School of Basic Medical Sciences, Guangzhou Medical University, Guangzhou, P. R. China

**Keywords:** air humidity, microdroplets, reactive oxygen species, SARS-CoV-2

## Abstract

Water droplets containing the SARS-CoV-2 virus, responsible for coronavirus 2019 transmission, were introduced into a controlled-temperature and -humidity chamber. The SARS-CoV-2 virus with green fluorescent protein tag in droplets was used to infect Caco-2 cells, with viability assessed through flow cytometry and microscopic counting. Whereas temperature fluctuations within typical indoor ranges (20°C–30°C) had minimal impact, we observed a notable decrease in infection rate as the surrounding air’s relative humidity increased. By investigating humidity levels between 20% and 70%, we identified a threshold of ≥40% relative humidity as most effective in diminishing SARS-CoV-2 infectivity. We also found that damage of the viral proteins under high relative humidity may be responsible for the decrease in their activity. This outcome supports previous research demonstrating a rise in the concentration of reactive oxygen species within water droplets with elevated relative humidity.

## Introduction

The seasonal variation of respiratory viral diseases, such as influenza, have long been recognized with the incidence of infection waxing in the wintertime and waning in the summertime (Moriyama *et al.*, 2020). Evidence of the same infectious behavior for the SARS-CoV-2 virus, the virus responsible for the coronavirus 2019 (COVID-19) pandemic, has also been presented, as shown in Figure S1 in the Supplementary Material (Shamsa *et al.*, 2023), although the exact behavior in time has been difficult to determine because of the emergence of virus variants and the uneven preventative public health measures taken (Townsend *et al.*, 2023).

What causes this seasonality is not yet fully established, with three different theories put forward: the effect of climate conditions on (1) host resistance to infection, (2) host social behavior, and (3) virus survivability (Price *et al.*, 2019). Increasingly, evidence has pointed to the role of relative humidity of indoor air, with a strong correlation found for increased virus survival with low relative humidity (Choi *et al.*, 2021; Liu *et al.*, 2021; Pineda Rojas *et al.*, 2021; Wang *et al.*, 2021; Nieto-Caballero *et al.*, 2022; Oswin *et al.*, 2022; Park *et al.*, 2022; Ravelli and Martinez, 2022; Verheyen and Bourouiba, 2022; Yin *et al.*, 2022). It has been proposed that the change in indoor relative humidity with seasons is caused by the fact that we heat the indoor air in the colder months to keep the occupants warm, which dries out the indoor air (Lowen and Steel, 2014). Nevertheless, because the cause of this correlation between airborne viral infection and relative humidity has not been established, the control of relative humidity has not been adopted as a public health policy. This study advocates for a change in this viewpoint.

Several theories have been advanced to explain how relative humidity affects virus survivability. These involve pH change at the droplet surface, changes in strength of the virus protein structure, and efflorescence in which salt or carbohydrate coat and protect the virus upon evaporation which is accelerated at lower relative humidity (Nieto-Caballero *et al.*, 2022; Oswin *et al.*, 2022). Recent work has also shown that as the relative humidity increases, the concentration of reactive oxygen species (ROS) in water droplets increases, such as the hydroxyl radical (OH) and hydrogen peroxide (H_2_O_2_) (Dulay *et al.*, 2021; Mofidfar *et al.*, 2024). Previous work has demonstrated that ROS in water droplets can act as a bactericide with high effectivity, much larger than if it were only caused by the presence of H_2_O_2_ (Dulay *et al.*, 2020). The present study demonstrates that water droplets surrounded by air at different relative humidity levels affect the infectivity of SARS-CoV-2 that is contained inside water droplets. Specifically, we show that as the relative humidity of the air is raised to 40% or more, there is a noticeable decline in the infection rate of SARS-CoV-2. The public health implications of these findings are briefly discussed.

## Methods

### Cell culture

An immortalized cell line of human colorectal adenocarcinoma cells with overexpression of SARS-CoV-2 viral N protein (Caco-2-N) were maintained in Dulbecco’s modified Eagle medium (DMEM; Gibco, China) supplemented with 10% (vol/vol) fetal bovine serum (FBS), and 50 IU/ml penicillin/streptomycin in a humidified 5% (vol/vol) CO_2_ incubator at 37°C. This cell lines were tested negative for mycoplasma.

### SARS-CoV-2 GFP/ΔN virus production

A *trans*-complementation system for SARS-CoV-2 including viral nucleocapsid gene (N) deficiencies SARS-CoV-2 virus, and Caco-2-N cell line that overexpresses N protein was used to produce SARS-Cov-2 in a biosafety level-2 (BSL-2) cell culture system, which recapitulates authentic viral infection and replication but without virulence. In detail, Caco-2-N cells were infected with P0 virus of SARS-CoV-2 GFP/ΔN to amplify the virus. Briefly, Caco-2-N cells were first spread in a 6-well plate, and after the cells were adhered to the wall, the medium was changed to a maintenance medium (DMEM with 2% FBS and no P/S), followed by the addition of P0 virus to the wells of a cell plate that was transferred to an incubator for virus amplification. The cell supernatant was harvested after 48 h and centrifuged to remove impurities before ultracentrifuging to obtain the P1 virus.

### Microdroplet generation and collection

The overall experiment was conducted in a closed environmental chamber with a controlled relative humidity in the range of 20%–70% and controlled temperature in the range of 10°C–40°C. Microdroplets were generated by spraying the aqueous viral suspension at a rate of 5 μl/min through a 100 μm inner diameter fused silica tubing with 120 psi air coaxial sheath gas in a sealed chamber at 25°C under relative humidity in range from 20% to 70%. The sprayed viral microdroplets were collected in a Petri dish containing PBS solution filtered by 0.22 μm syringe filter, which was located 15 cm from the nebulizer.

### Nanoparticle tracking analysis (NTA)

The harvested virus solution was first transferred to a biosafety cabinet, then 100 μl was removed and diluted fivefold with PBS to form the sample. All samples were measured by Nanosight NS300 (Malvern Pananalytical Ltd., Malvern, UK) at 25°C. Each sample was measured for 60 s and repeated five times, and finally the mean value was calculated.

### Infection by virus

After nanoparticle tracking analysis (NTA) measurement of the viral content of the collected viral solution, the same amount of virus was used to infect the 48-well plate that had been previously spread with Caco-2-N cells. Positive control wells were filled with the same amount of virus from untreated stock virus solution and negative controls were filled with the same volume of virus-free PBS. The plates were then incubated at 37°C with 5% CO_2_ for 48 h.

### Confirmation of viral activity by flow cytometry

After 48 h of virus infection, cells were washed in cold PBS and digested by trypsin (Thermo Fisher Scientific, Waltham, MA, USA) before collected in 1.5 ml microcentrifuge tubes. The percentage of virus-infected cells and fluorescence intensity were analyzed by FACS by using CytoFLEX (Beckman Coulter, Brea, CA, USA). Data were analyzed using FlowJo V10 software (Becton Dickinson, Franklin Lakes, NJ, USA).

### Immunofluorescence assay

After 48 h of virus infection, cell culture plates were transferred to an inverted fluorescence microscope CKX53 (Olympus, Tokyo, Japan) or ZEISS LSM 900 confocal laser scanning microscope (ZEISS, Jena, Germany), and photographed using the accompanying computers. Analysis of fluorescence light intensity was accomplished using ImageJ software.

### Western blot

Viruses contained in the collected viral solution after nebulization were quantified by the numbers of viral particles using the NTA test, and the same amount of virus particles were ultracentrifuged. A lysis buffer (50 mM Tris–HCl, pH 7.4, 300 mM NaCl, 1% Triton X-100, 5 mM EDTA, and 10% glycerol) containing a protease inhibitor (10%, Roche, 04693116001) were used to resuspend the viruses. Lysates were electrophoresed on 10% SDS-polyacrylamide gel electrophoresis (PAGE) and transferred to a polyvinylidene fluoride (PVDF) membrane. Nonspecific bands were blocked by 5% skim milk for 2 h and then incubated with SARS-CoV-2 spike protein mAb (Cell Signaling Technology, Boston, MA, USA), SARS-CoV-2 envelope protein antibody (Cell Signaling Technology, Boston, MA, USA), and SARS-CoV-2 membrane polyclonal antibody (Abnova, Taipei, China) at 4°C overnight. Protein bands were visualized using a standard enhanced chemiluminescent substrate (Epizyme, Cambridge, MA, USA).

### Gel electrophoresis

After determining the RNA content of the extracted nucleic acids solution, the same amount of RNA was removed for further use. A 0.9% agarose gel was prepared by dissolving agarose powder (Invitrogen, Thermo Fisher Scientific, Sunnyvale, CA, USA) in tris-acetate (TAE) buffer. Confirmed extracted nucleic acids solution with the same RNA amount were mixed with loading buffer (Thermo Fisher Scientific, Sunnyvale, CA, USA) before electrophoresed using 100 V for 60 min. Nucleic acid mobility was visualized using a GelDoc Go (Bio-Rad, Hercules, CA, USA).

### ROS concentration

A H_2_O_2_ standard solution reacted with 0.1 M potassium titanium oxalate (PTO) solution in equal volume, and the absorbance at 400 nm was determined by UV–Vis spectroscopy. Then, by varying the H_2_O_2_ concentration, a standard absorption curve was constructed. The samples (sprayed DI water microdroplets) were collected at the different relative humidity levels (20%–70%), reacted with 0.1 M PTO solution in equal volume, and the absorbances at 400 nm were determined. The PTO responds not only to H_2_O_2_ but other reactive oxygen species, such as OH.

## Results and discussion

**Figure 1.**
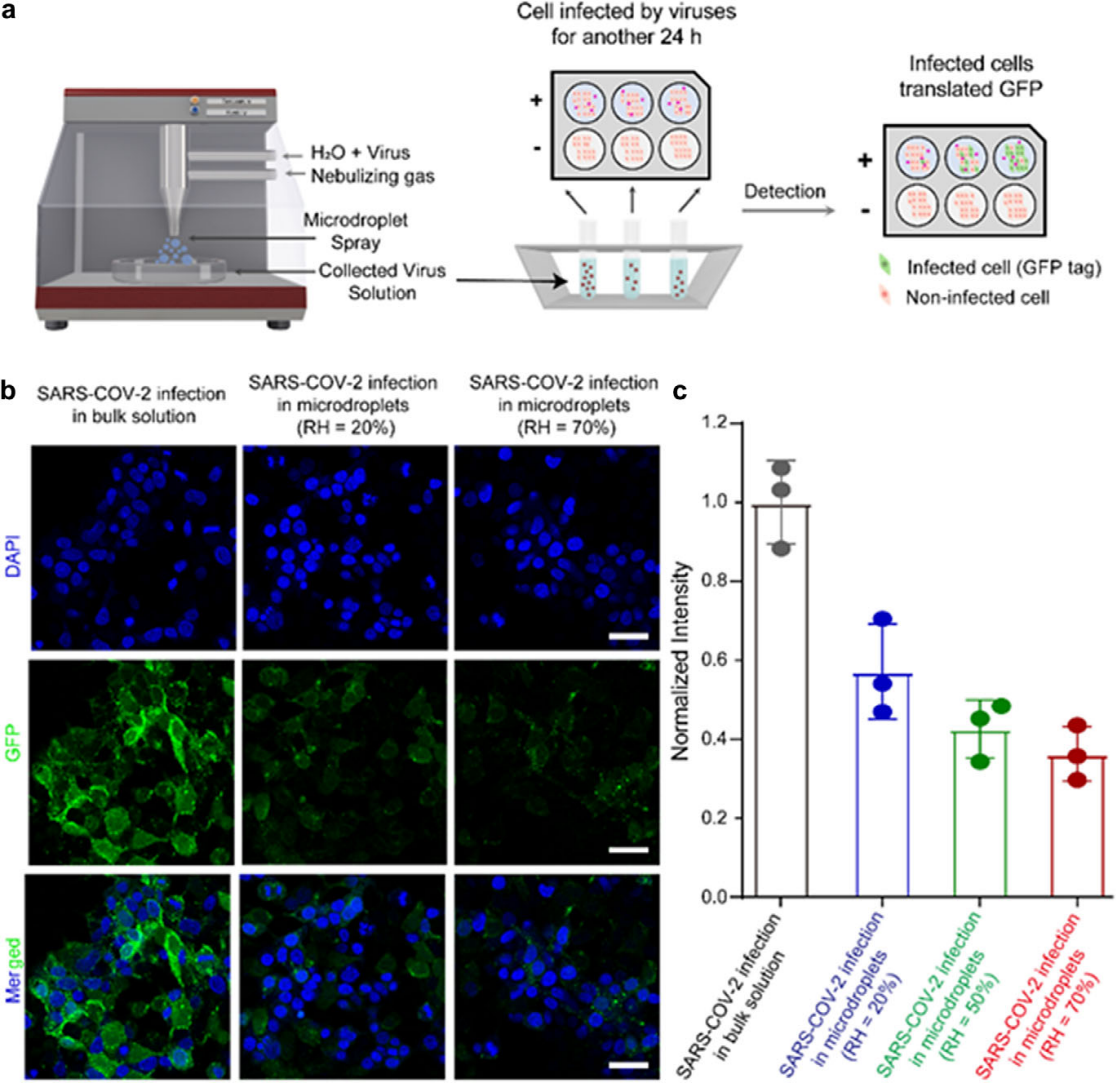
(*a*) Schematic image of the experimental setup of viral microdroplets generation in a sealed chamber. (*b*) Immunofluorescence image of Caco-2 cells infected by SARS-CoV-2 in bulk suspension, obtained from microdroplets collected under relative humidity (RH) of 20% and 70%, respectively, at 25°C. The scale bars are 40 μm. (*c*) Corresponding fluorescence intensity of infected Caco-2 cells.

To examine the effect of humidity on the activity of SARS-CoV-2 in microdroplets, we atomized the GFP-tagged virus-containing suspension in a sealed chamber and collected the viral microdroplets at 25°C under relative humidity of 20% and 70%, respectively (as shown in Figure 1*a*). We confirmed the virus concentration in each of the collected suspensions by NTA analysis and adjusted them to the same level. The collected viruses were then used to infect Caco-2 cells. We compared the differences in viral activity in three samples, which are the viruses in bulk aqueous suspensions, and in microdroplets collected at 20% and 70% relative humidity, by assessing the cellular infectivity. As shown in Figure 1*b*, Caco-2 cells were first observed with 4′,6-diamidino-2-phenylindole (DAPI). The cells with green fluorescence were infected by SARS-CoV-2. It is evident that the virus activity in the bulk aqueous suspension is higher than that in the microdroplets. More importantly, the activity of viruses collected at 70% relative humidity is lower than that of viruses collected at 20% relative humidity. We then analyzed the corresponding fluorescence intensity of infected Caco-2 cells using ImageJ software. As shown in Figure 1*c*, the intensity of the viruses in microdroplets decreased with the rise of ambient humidity. Therefore, we found that the activity of viruses in aqueous microdroplets changed with the change in ambient humidity, and the trend of the change was consistent with the previous reports of COVID-19 transmission (Ma *et al.*, 2021).

**Figure 2.**
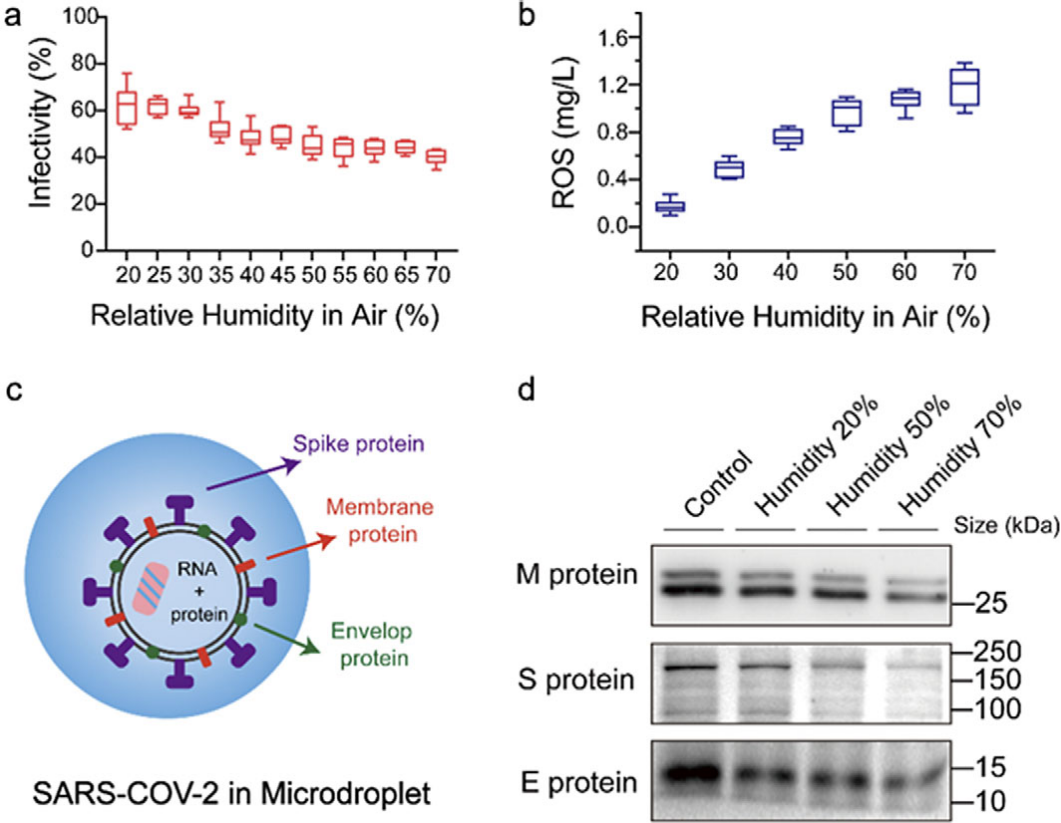
Variation with relative humidity of air surrounding microdroplets of (*a*) viral infectivity and (*b*) ROS concentration as measured by potassium titanium oxalate. (*c*) Schematic image of SARS-CoV-2 virus in a microdroplet. (*d*) Western blot analysis of membrane, spike, and envelope proteins of SARS-CoV-2 virus in microdroplets collected under different relative humidity levels.

We also examined how the viral activity changed with temperature (see Figure S2 in the Supplementary Material) but found little change over the range of 20°C to 40°C. We further analyzed the change in the infectivity of the viruses in the microdroplets as the relative humidity was varied. As shown in Figure 2*a*, the viral infectivity is 40% at 70% relative humidity and 62% at 20% relative humidity. When the humidity was higher than 60%, the decrease in virus infectivity was maintained at about 40%.

Previous work reported that ROS can be spontaneously generated in microdroplets (Lee *et al.*, 2019; Xia *et al.*, 2023). Therefore, we speculated that the change of viral activity in microdroplets may be related to ROS in microdroplets. To verify our conjecture, we tested the concentration of ROS in microdroplets at different humidity levels as shown in Figure 2*b*. As the relative humidity increases from 20% to 70%, the concentration of ROS increases from 0.2 to 1.2 mg/L.

Given that the distance between the nebulizer and the collector in our experimental design was only 15 cm, the flight time of the droplets in the air was less than 10 ms. Thus, the data presented refer to what happens at short times, such as face-to-face contact of a healthy person with a sick person. It might be expected that the effect of relative humidity on viral infectivity would be even more pronounced for longer times before droplet evaporation.

Recent work suggests that change in viral activity in microdroplets may be related to evaporation of microdroplets and diffusion of carbon dioxide into the microdroplets in air (Nieto-Caballero *et al.*, 2022; Oswin *et al.*, 2022). The evaporation of the droplets and the diffusion of carbon dioxide can hardly explain the phenomena we observed because our data refer to droplets that only travel for such short times.

Destruction of viral proteins was confirmed through Western blot as shown in Figure 2*c* *,* *d*. Damage of viral membrane, spike and envelope proteins rises as humidity increases. While destruction of RNA can hardly be observed as the increase of humidity as shown in Figure S3 in the Supplementary Material. These results indicate that spontaneous generation of ROS in microdroplets disrupts proteins on the membrane of SARS-CoV-2, and thus leads to a decrease in viral infectivity. In addition, we have also used a standard ROS initiator (H_2_O_2_) to treat the virus solution and the results of the NTA test (see Figure S4 in the Supplementary Material), and we found that Coomassie brilliant blue staining (see Figure S5 in the Supplementary Material), and BCA assay (see Figure S6 in the Supplementary Material) indicated that the viral particles remain constant, but viral proteins gradually degrade with increased ROS. Therefore, the results further confirm that the change of ROS concentration in microdroplets in response to humidity changes may be one of the factors affecting viral infectivity. Our results suggest that the initial activity of viruses that enter the air by exhalation from a sick individual may exhibit seasonal variations caused by changes in the relative humidity of the air surrounding water droplets containing SARS-CoV-2.

## Conclusion

In this study, we found that SARS-CoV-2 viral infectivity in microdroplets decreases as the relative humidity of air surrounding microdroplets increases. Moreover, the decrease in viral infectivity tends to be nearly constant when the humidity is greater than 60%. This trend is basically consistent with the observation that ROS concentration in microdroplets rises with humidity. This phenomenon can be attributed to the damage of proteins on the membrane of virus caused by ROS in microdroplets. Our findings provide a new perspective for understanding the seasonal spread of COVID-19 disease, and it is certainly expected to explain the seasonal variation observed for other infectious viral respiratory diseases. These findings strongly suggest the need to control indoor relative humidity for reducing the spread of viral respiratory infections, such as COVID-19.

## Supporting information

Liu et al. supplementary material Liu et al. supplementary material

## Data Availability

All data may be requested from the corresponding authors.

## Supplementary material

The supplementary material for this article can be found at http://doi.org/10.1017/qrd.2024.7.
