# Supplementary material for: As air relative humidity increases, infectivity of SARS-CoV-2 decreases within water droplets
Source: QRB Discov. 2024 Aug 29;5:e6. doi: 10.1017/qrd.2024.7 (PMC11649373; doi:10.1017/qrd.2024.7)
Supplement: Liu et al. supplementary material [file S2633289224000073sup001.pdf]

Supporting Information

As Air Relative Humidity Increases, Infectivity of SARS-CoV-2 Decreases Within Water Droplets

Yu Liu[1*], Lei Cao[1*], Yu Xia[2, 3], Pan Pan[4], Lang Rao[1†], Bolei Chen[2†], Richard N. Zare[3†]

[1]Institute of Biomedical Health Technology and Engineering, Shenzhen Bay Laboratory, Shenzhen, Guangdong, 518132, P. R. China

[2]Hubei Key Laboratory of Environmental and Health Effects of Persistent Toxic Substances, School of Environment and Health, Jianghan University, Wuhan, 430056, P. R. China

[3]Department of Chemistry, Stanford University, Stanford, CA 94305, USA

[4]School of basic medical sciences, Guangzhou medical university, Guangzhou, Guangdong, 510182, P. R. China

[*]These authors contributed equally

†To whom correspondence may be addressed. Email: lrao@szbl.ac.cn, bl_chen@jhun.edu.cn, zare@stanford.edu

Table of Contents

Figure S1 Daily COVID-19 incidences in the USA over the 2020–2022 seasonal years..................S3

Figure S2 Variation of virus infectivity as a function of temperature for different relative humidity levels..S4

Figure S3 RNA of SARS-CoV-2 virus in microdroplets collected under different relative humidity levels………S5

Figure S4 The NTA results of SARS-CoV-2 treated under different concentrations of H_2O_2 ….S6

Figure S5 The Coomassie brilliant blue staining results of SARS-CoV-2 treated under different concentrations of H_2O_2 ……………………………………………………………………………………………S7

Figure S6 The BCA protein assay results of SARS-CoV-2 treated under different concentrations of H_2O_2 ………S8

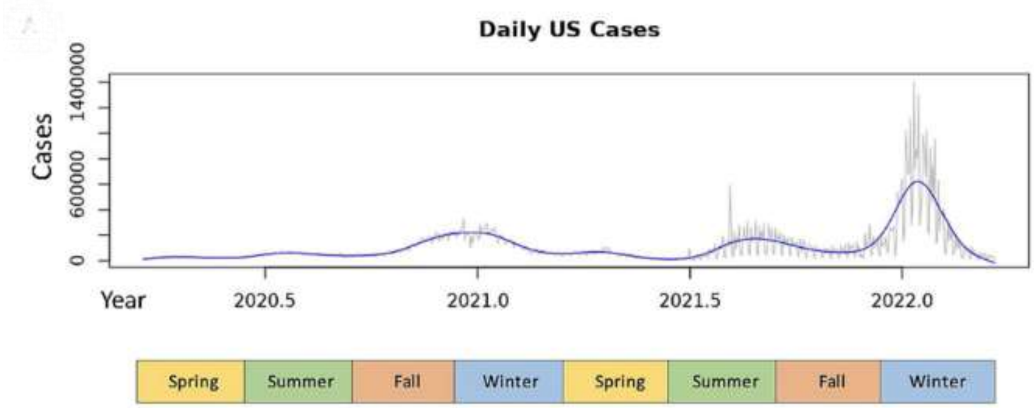

Figure S1. Daily COVID-19 incidences in the USA over the 2020–2022 seasonal years. The gray line represents the daily measures for the raw data time series, and the blue line is the smoothed cubic spline fit to the time series. Taken from Shamsa, Shamsa, and Zhang, 2023 with permission.

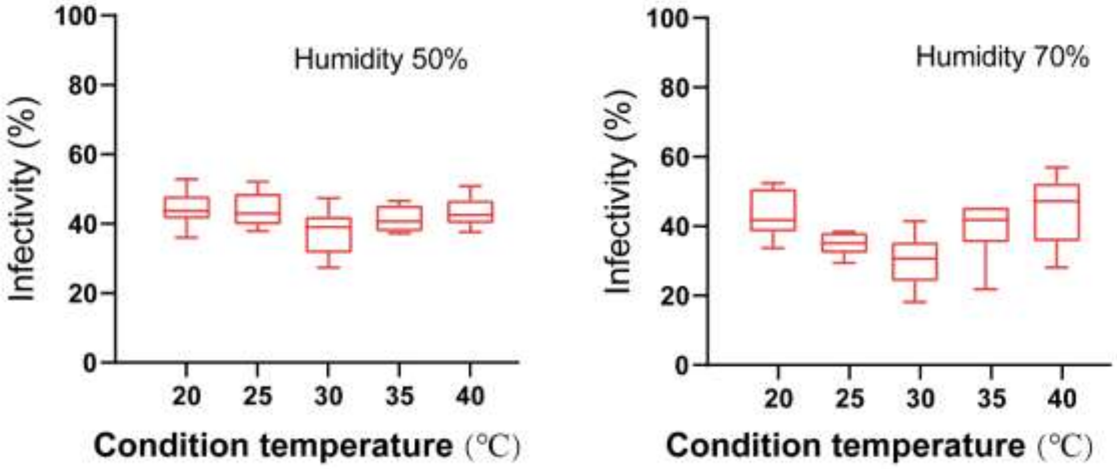

Figure S2. Variation of virus infectivity as a function of temperature for different relative humidity levels.

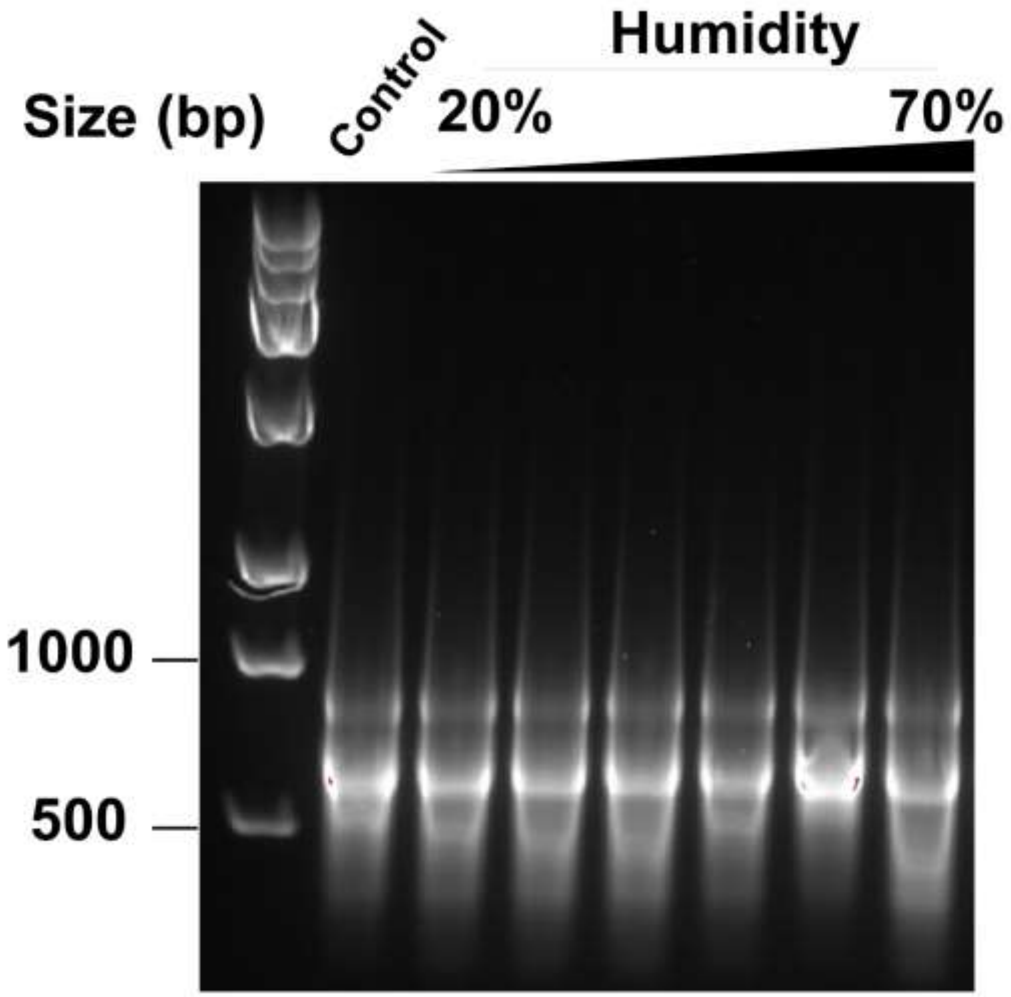

Figure S3. RNA of SARS-CoV-2 virus in microdroplets collected under different relative humidity levels.

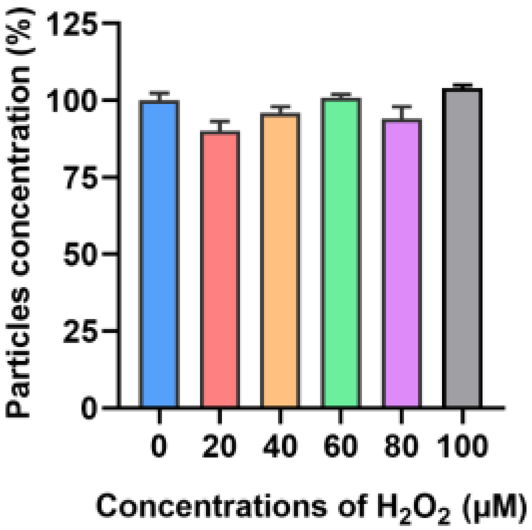

Figure S4. The NTA results of SARS-CoV-2 treated under different concentrations of H_2O_2.

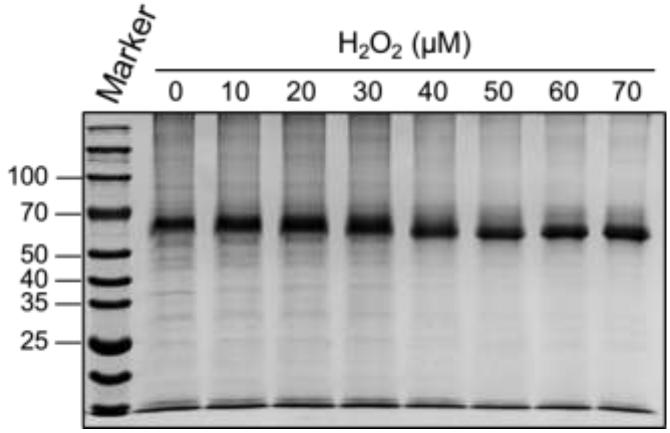

Figure S5. The Coomassie brilliant blue staining results of SARS-CoV-2 treated under different concentrations of H_2O_2.

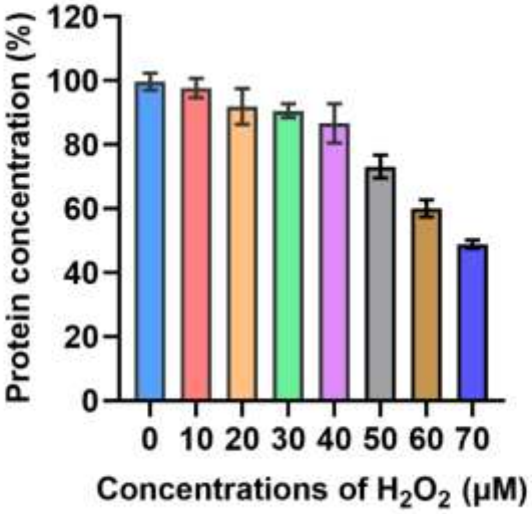

Figure S6. The BCA protein assay results of SARS-CoV-2 treated under different concentrations of H_2O_2.
