# Supplementary material for: As air relative humidity increases, infectivity of SARS-CoV-2 decreases within water droplets
Source: QRB Discov. 2024 Aug 29;5:e6. doi: 10.1017/qrd.2024.7 (PMC11649373; doi:10.1017/qrd.2024.7)
Supplement: Peer review history

# Editor report: Decision: As air relative humidity increases, infectivity of SARS-CoV-2 decreases within water droplets — R0/PR2

**DOI:** 10.1017/qrd.2024.7.pr2

**Affiliations:** Chemistry, Chalmers University of Technology, Sweden

I am happy to invite authors to revise their manuscript in the light of the reviewers' comments. In particular I find it important to comment on critical points raised by two reviewrs and complement the manuscript with the information asked for.

# Editor report: Decision: As air relative humidity increases, infectivity of SARS-CoV-2 decreases within water droplets — R1/PR4

**DOI:** 10.1017/qrd.2024.7.pr4

**Affiliations:** Chemistry, Chalmers University of Technology, Sweden

No accompanying comment.
